# Micromagnetic simulations with periodic strayfield calculation of soft magnetic composite-materials

**DOI:** 10.1038/s41598-025-01881-8

**Published:** 2025-05-22

**Authors:** Amil Ducevic, Florian Bruckner, Claas Abert, Christoph Vogler, Dieter Suess

**Affiliations:** 1 Physik funktioneller Materialien, Universität Wien, Kolingasse 14-16, Wien, 1090 Austria; 2 Huawei Industrial Base Bantian Longgang, Huawei Technologies Co., Ltd., Shenzhen, 518129 China

**Keywords:** Electronic and spintronic devices, Applied physics, Ferromagnetism, Magnetic properties and materials

## Abstract

This work provides a complete numeric framework, with which soft magnetic composite materials can be numerically analyzed through a combination of two distinct numerical methods. Key soft magnetic properties connected to energy efficiency, namely the permeability and the energy-loss can be predicted. The latter consists of different contributions. These contributions are treated separately in our work and therefore 2 decoupled numerical models are required to fully encompass these materials. Experimentally established concepts with the aim to improve magnetic properties for soft magnetic materials have been simulated, resulting in trends that agree well with the experimentally measured improvements. Moreover, we analyze the distinct loss contributions-an uncommon practice in experimental physics-providing valuable insights into the working principles of these materials, and therefore understanding which contributions become dominant under which conditions. Furthermore measurements of iron powders were conducted, in order to compare the numerical simulations with experimental data.

## Introduction

2022 showed a worldwide increase in energy consumption by 1.11%^1^, if compared to the prior year, this yields a total energy usage of 178.389 TWh, which marks a record in yearly global energy consumption. 2022 is not an outlier in this regard, with the exception of 2019 and 2009, since 1990 the trend of the global power consumption is growing with each year. As one way to combat this the EU has committed itself to reduce the energy consumption by 32.5%^2^ until 2030, as compared to 2007.

Electrical energy has accounted for roughly 20%^3^ of the worldwide energy consumption in 2022 and is forecast to increase to 50% until 2050^3^. Nearly all means of generating electrical energy, whether they are based on fossil fuels or renewable energies are based on generators powered by turbines, with the only exception being solar power. Soft magnetic materials are vital to generators and essentially every electric device that is currently being used, therefore we believe strongly that a simulation model which can predict the performance of soft magnets would be of great value. This work is focused on modeling a specific kind of soft magnet, however our methods could be used to study and improve different kinds of soft magnets as well.

A subcategory within soft magnetic materials are soft magnetic composites(SMCs). The market of SMCs in 2019 was approximated at 19.23 billion USD^4^ and is estimated to grow by 2029 to a value of 80 billion USD^5^. These materials have emerged in the 1990s and consist of a ferromagnetic powder, with particle sizes which range from  1 to 100 ${\upmu }\hbox {m}$, which is coated by an electrical insulating material. The advantage of using an insulated powder, as opposed to a bulk material, is the decreased eddy-current loss at medium and high frequencies. The obvious disadvantage is that the permeability decreases, due to the airgap between the ferromagnetic particles. Until now there have been numerous studies^6–8^, which attempt at modeling SMCs utilizing numeric methods. The work of Arzbacher et al.^6^ is focused on modeling the demagnetizing field through Maxwells equations based on tomography data of the SMCs microstructure, while Weir et al.^7^ establish an analytical model in order to predict the permeability of SMCs. Bruckner et al.^8^ come closest to the study at hand, since it is also calculating SMCs utilizing micromagnetics. However the study in question, is a proof of concept, for the calculation of the demagnetization fields with PBCs and does not attempt to realistically depict the shape, the size, the material parameters or results which are comparable with commercially available SMCs. Within this work we perform efficient micromagnetic simulations utilizing true periodic boundary conditions proposed by Bruckner et al.^8^, along with numerically solving Maxwells equations, in order to realistically model SMCs and to optimize the SMC microstructure for low energy losses and high permeability.

We define the relative magnetic permeability $\mu _r$ as

1 $$\begin{aligned} \mu _r = \mu /\mu _0 = \frac{1}{\mu _0}\frac{dB}{dH}, \end{aligned}$$

with $\mu$ being the magnetic permeability, $\mu _0$ the magnetic permeability in vacuum, *B* the magnetic flux density, and *H* the applied external magnetic field. In this work $\mu _r$ is always evaluated at $H=0$. In the following $\mu _r$ will be referred to as the permeability.

The energy loss of SMCs can be split up into different contributions, the majority of literature^9–12^ available agrees on the definition as follows

2 $$\begin{aligned} W(f)=W_{\text {hyst}}+W_{\text {eddy}}(f)+W_{\text {res}}(f), \end{aligned}$$

$W_{\text {hyst}}$ is the static hysteresis loss, which arises from irreversible domain-wall motion. $W_{\text {eddy}}$ is the classic eddy current loss, the cause of which are electrical resistance losses within the material caused by a magnetic field which alternates with the frequency *f*, one can further separate this into inter- and intra-particle losses, meaning losses which arise from eddy currents within the particle and losses attributed to defects in the coating. Lastly $W_{\text {res}}$ is the residual loss, which is usually defined as all other losses of unclear origin.

In this work, we have chosen to examine SMCs utilizing computational micromagnetism and Maxwell solvers. The micromagnetic model can resolve the structure of magnetic domains and domain walls, this yields accurate static hysteresis computation, with which $W_{\text {hyst}}$ can be predicted. Another feature of micromagnetics is its ability to resolve the time-dependent magnetic switching processes and domain-wall movements. This is crucial due to the fact that applied frequencies of the external field are in the high-frequency regime($\sim 1$ MHz), where the residual loss $W_{\text {res}}$ becomes relevant. A part of the residual losses originate in the increase of the hysteresis, which results from the fact that the magnetization lags behind the applied magnetic field due to its high frequency, we will refer to this as $W_{\text {dyn}}$. This means that if one were to calculate the time-dependent hysteresis with the chosen micromagnetic-code, part of the total power loss is given, by calculating the area of the hysteresis. We call this the micromagnetic power-loss and define it as follows

3 $$\begin{aligned} P_m&= f\cdot W_{m}=f\cdot (W_{\text {hyst}}+W_{\text {dyn}})\nonumber \\&= f\cdot \oint {B(H)dH} \le f \cdot (W_{\text {hyst}}+W_{\text {res}}). \end{aligned}$$

From here on out we will refer to $P_{m}$ as micromagnetic power-loss. Although our micromagnetic model does not predict eddy current losses, they can be calculated using Maxwell solvers, which effectively solve Maxwells equations. These solvers enable us to accurately compute the electric currents induced by an alternating magnetic field, allowing us to determine the eddy-current loss with the following equation

4 $$\begin{aligned} P_e=\frac{1}{2\sigma }\int |\mathbf {J(\textbf{x})}|^{2}dA, \end{aligned}$$

where $\sigma$ is the conductivity and $\textbf{J}$ is the current density.

## Micromagnetic theory

This section aims to give a short overview over both the micromagnetic theory, which lies at the core of the simulation work at hand. Firstly the individual micromagnetic energy contributions will be shortly discussed, with emphasis on how the inclusion of periodic boundary conditions(PBCs) alter the strayfield-calculation, as was already outlined in Ref.^8^. PBCs are essential in order to accurately simulate SMCs, due to the fact that the magnetic samples are very large, typically in the millimeter-regime, however only a small part of the structure can be simulated, since the application range of micromagnetism is in the nano- to micrometer regime. Simply truncating the magnetic domain would lead to a strong shape anisotropy and therefore to a much smaller permeability, and since the shape anisotropy is scale invariant, the actual size of the truncated geometry does not matter. For the same reason also pseudo periodic boundary conditions, as commonly used by many micromagnetic codes, will not produce correct results. All micromagnetic simulations in this work will be performed utilizing a GPU-accelerated finite difference code called $\mathbf {magnum.np}$^13^, which will dramatically reduce the computation time.

**Fig. 1:**
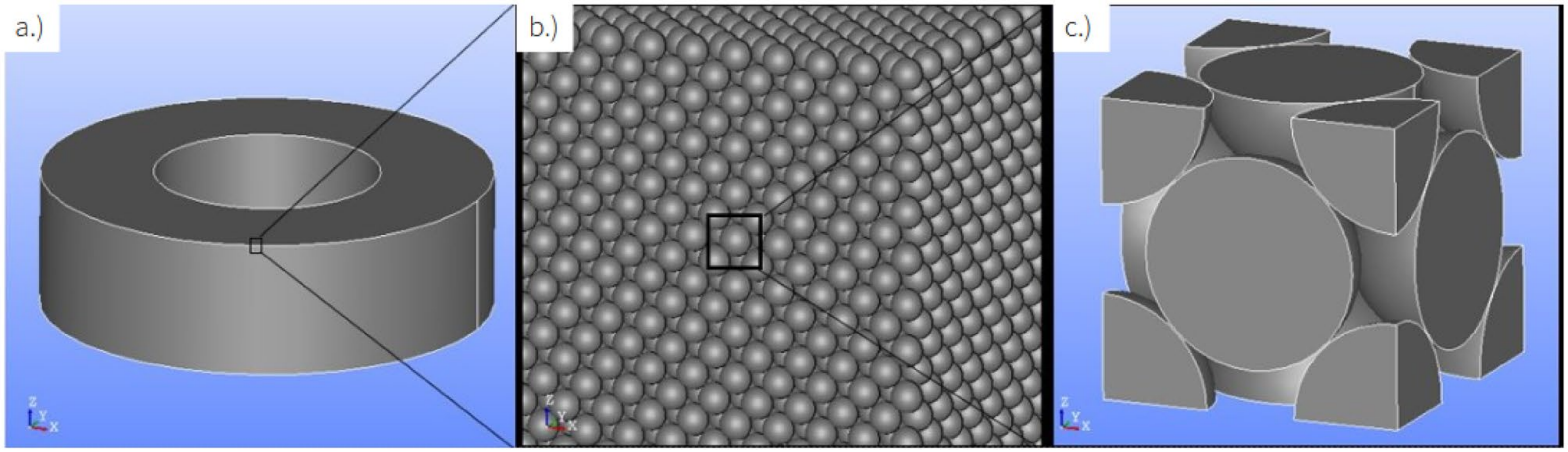
A schematic of the simulation cell and how it relates to the simulated lattice and the magnetic core (**a**) A toroidal magnetic core, as it would be produced from SMCs. (**b**) Zooming into the core, reveals the, oversimplified model, that the iron based particles form a perfect fcc-lattice, where the particles are not exchanged coupled but have an interparticle layer between them (**c**) The unit-cell of a fcc-structure, as it is utilized in the micromagnetic simulation with PBCs.

The energy of a ferromagnet highly depends on the external field $\textbf{H}_{\text {zee}}$, therefore the simulations will employ the Zeeman-Energy. A SMC which is pressed into a transformer core, as can be seen in Fig. 1a, is subject to an external field provided by the primary coil. Therefore the external field within the simulation will have the form

5 $$\begin{aligned} \textbf{H}_{\text {zee}}=\textbf{H}_{\text {ext}} \cdot sin(\omega t + \phi ), \end{aligned}$$

where $\mathbf {H_{\text {ext}}}=(H_{\text {ext}},0,0)$ is the amplitude, $\omega =2\pi f$ is the angular frequency and $\phi$ is the phase-difference. Since this is a local energy contribution, the calculation and numerical implementation doesn’t change with PBCs, when compared to open-boundary conditions.

Since iron has a cubic lattice symmetry, with a body-centered cubic structure, and most SMCs are iron based powders the simulations will utilize cubic anisotropy. Similarly to the Zeeman energy, the anisotropy energy is a purely local energy contribution, therefore the computation remains unaltered.

Every simulation cell is exchange coupled to its nearest neighboring cells through the exchange field, therefore the calculation changes slightly when PBCs are taken into account. Periodic boundary condition take the initial simulation box and replicate it infinitely in all directions, this means that the simulation cell on an edge surface is exchange coupled with the corresponding cell on the opposing edge surface, when evaluating discretized differential operators.

The demagnetizing field is notoriously the most cost-heavy energy-contribution to calculate, due to the fact that it is a long range interaction. Usually one solves

6 $$\begin{aligned} \Delta u = \nabla \cdot \textbf{M}, \end{aligned}$$

with *u* being the scalar potential of the demagnetizing field and $\textbf{M}$ the magnetization. A standard method to solve (6) is to use the FFT and to apply zero padding in order to solve for the open boundary problem^14^ and for the scalar potential^15^. However since the demagnetizing energy is a long range interaction, we again have to alter the computation to fit the PBCs. Some standard micromagnetic codes use pseudo PBCs where the summation is truncated after a finite number of periodic images^16^. We will employ the methods of Ref.^8^ in order to calculate the demagnetizing field with true PBCs.

## Maxwell solver theory

To calculate the eddy-current-losses we have to utilize a different model than our micromagnetic framework. In order to calculate the current density $\textbf{J}$ we are going to solve Maxwells-equations numerically. However we will not discuss here in detail how we are bringing the equations into the weak formulation to solve them utilizing finite-elements. We will focus here on explaining the simulation model, and how we achieve periodic boundary conditions with the Maxwell–Solver. We have to utilize a different method for PBCs, because we were not able to unify the Fourier-based^8^ approach with the vectorfield-formulation. Therefore we want to implement periodic boundary conditions by using symmetry boundary conditions inspired by Ref.^17^. Therein a matrix of magnetic cubes is calculated, but instead of modeling all of the cubes, only one 1/8-cube is considered with symmetry conditions. Meaning that effectively the calculated space dependent, magnetic flux density of the 1/8-cube should be equivalent to the result for that part of a whole cube which is surrounded on all sides by cubes of the same size. The symmetry boundary conditions are

7 $$ \begin{aligned} &\textbf{B}\cdot \textbf{n}=0 \text { : On the 4 side surfaces} \\&\textbf{B}\times \textbf{n}=0 \text { : On the top  \&  bottom surfaces}, \end{aligned} $$

with *n* being the orientation vector of the surface in question. The boundary conditions in (7) mean that $\textbf{B}$ has to be tangential to the side planes and normal to the bottom and top plan. The simplest way to implement this is to utilize the reduced form of the Maxwell equations within *Comsol*.

8 $$\begin{aligned} \textbf{J}&=\nabla \times \textbf{H}, \\ \textbf{B}&=\nabla \times (\textbf{A}_b+\textbf{A}_r), \\ \textbf{J}&=\sigma \textbf{E}+j\omega \textbf{D}+\textbf{J}_e, \\ \textbf{E}&=-j\omega (\textbf{A}_b+\textbf{A}_r), \end{aligned}$$

where $\sigma$ is the electric conductivity, $\textbf{D}$ is the displacement current field, $\textbf{E}$ is the electric field and the magnetic vector potential $\textbf{A}=\textbf{A}_b+\textbf{A}_r$ is separated into a background field $\textbf{A}_b$ and the reduced field $\textbf{A}_r$. Due to this set of equations the symmetry conditions from (7) change to

9 $$ \begin{aligned} &\textbf{n}\times \mathbf {A_r}=0 \Rightarrow \textbf{B}\cdot \textbf{n} = 0\text { : On the 4 side surfaces}, \\&\textbf{B}\times \textbf{n}=0 \text { : On the top  \&  bottom surfaces}. \end{aligned} $$

The obvious limit of these boundary conditions is that symmetry is required, i.e. in this model we would not be able to apply the magnetic field in a direction that isn’t parallel to a coordinate axes. However for the study at hand this limitation is of no consequence.

## The simulation model

### Geometry

There are a wide variety of shapes when it comes to the magnetic powder, e.g. spheres^18–22^ , flakes^23–26^ or ribbons^27^. However the vast majority of ferromagnetic powders employed in SMCs are spherical, therefore we will also employ spherical particles. The ferromagnetic spheres were arranged in an fcc-structure. The particles are all separated by an interparticle layer, which is non-magnetic and non-conductive, therefore the particles are decoupled with regard to the exchange interaction. This gives us an upper threshold for the filling-factor of 74%, with the filling factor being the (volume-)fraction of magnetic material. The actual filling-factor in the simulation will be slightly reduced, due to the fact that we have to introduce a small gap between the individual particles. The filling factor used throughout all simulations in this work is 70%. Since we utilize PBCs we do not need to model an entire fcc structure, as is shown in Fig. 1b but can simply model a unit-cell with half and eighth spheres. This gives a cubic simulation box with six half-spheres centered at the 6 edge surfaces and eight eighth-spheres situated at each corner of the cube, which if repeated an infinite number of times, in all directions, gives a complete fcc structure. The micromagnetic model uses the simulation box as it is shown in Fig. 1c.

To ensure that the results of the eddy-current calculations are comparable with the micromagnetic model, it would be advantageous to simulate just the primitive cell of the fcc structure. However, this approach proved impractical because the simulation box in the eddy-current model cannot intersect magnetic material. If we cut through a region where current density flows-such as a half-sphere-the current density would form closed loops around the half-sphere. However, since the magnetic field $\textbf{H}$ is always applied in the z-direction, the current paths are constrained to the xy-plane. Therefore, cutting through the xy-plane does not interfere with the current paths and is permissible.

**Fig. 2:**
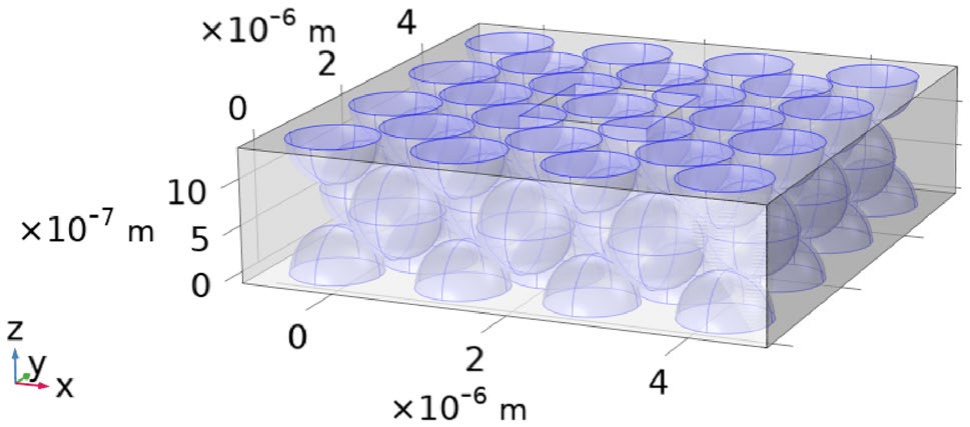
The geometry that was chosen for the Maxwell-solver model is the primitive cell of the fcc-structure repeated in a $3\times 3$ grid with the spheres being cut along the xy-plane. A cube is added in the center of the mesh, this cube has no physical meaning, but is solely there so that one could average over a central primitive fcc-cell.

The solution, shown in Fig. 2, involves repeating the primitive cell with whole spheres in a 3 $\times$ 3 grid, which is then sliced along the xy-plane. Additionally, a cube the size of the primitive cell is placed in the center of the model. This central cube does not represent any physical structure but is used for averaging purposes, to make the results comparable to the micromagnetic simulations. For example, when we report that $B = 100\,\text {mT}$ in an eddy-current simulation, this means that the z-component of the magnetic flux density, averaged over the central cube, equals 100 mT.

### Grains and particles

**Fig. 3:**
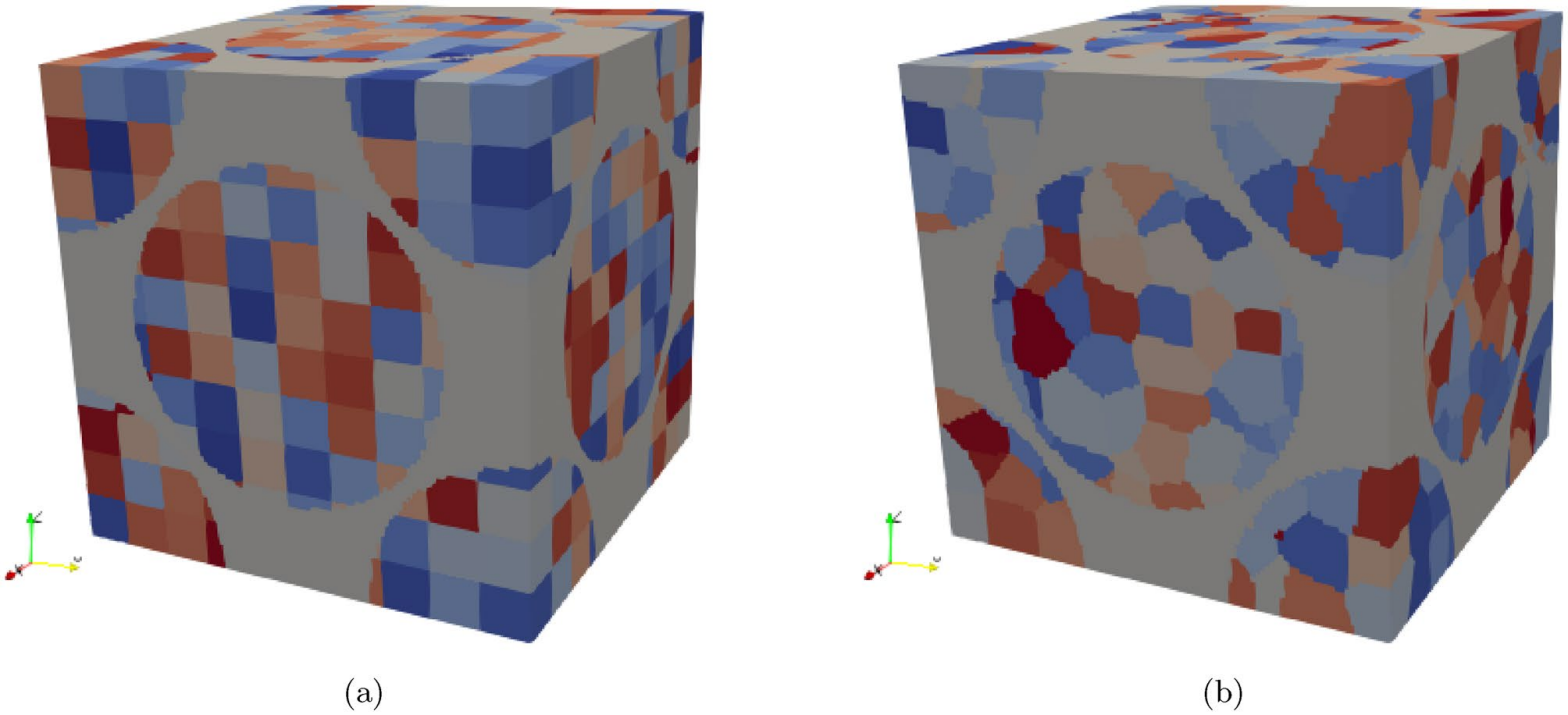
Two different implementations for the grain-distribution (**a**) Regular cuboid grains with equal volumes (**b**) Voronoi grains with unequal volumes.

Not only does the spacial distribution of the spheres matter, but the spheres also have a microstructure, which can be resolved by micromagnetics. The spherical iron particles consist of smaller grains which are exchange coupled, and have altering random easy axes in each grain. This only needs to be considered in the micromagnetic model, since Maxwells equations do not account for crystal anisotropy. In this work the axes will be chosen randomly, so that they are evenly distributed on the unit sphere, the three orthogonal easy axes are $[\textbf{e}_i,\textbf{e}_j,\textbf{e}_k]$. The entire simulation box is then divided into $n\times n\times n$ equally sized cubic grains, as can be seen in Fig. 3A. This gives the advantage that the dependence on grain-size is very easily controllable, since one can simply increase *n* in order to decrease the size of the grain, but the disadvantage is that it is not necessarily realistic. We will refer to a set of directions which is needed for one simulation as a seed of directions. Another possibility is to generate Voronoi cells as grains, as is shown in Fig. 3B.

### Simulation setup

**Fig. 4:**
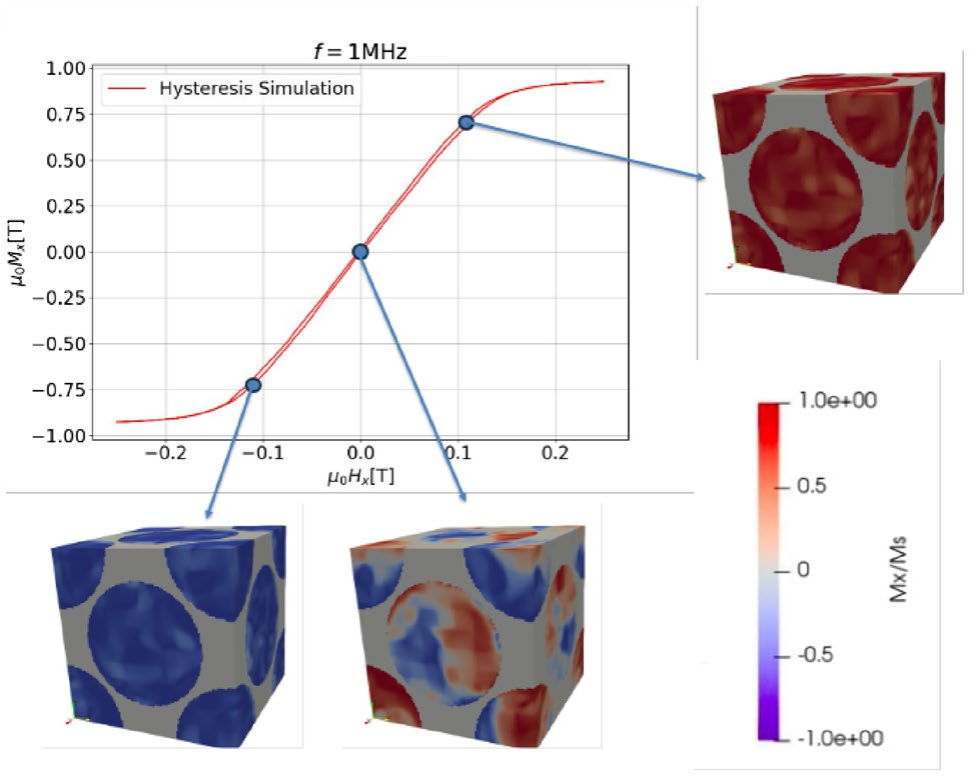
A hysteresis loop of a soft magnetic composite simulation is shown, along with the magnetization images at different field magnitudes.

In Tables 1 and 2 the parameters of the particle and the filler material are shown alongside the references for the respective values, for the two different simulation models. For the saturation polarization of the iron alloys we use $\mu _0 M_s=1.5\text {T}$, which is within the range of experimentally reported values^28^. Note that when $M_s$ is discussed, it is always supposed to mean the magnetic saturation of the magnetic material the spherical particles consist of. Since the micromagnetic simulation averages the magnetization over the entire simulation box, the hysteresis would not saturate at $M_s$, but at $M_{s,SMC}=\rho M_s$, where $\rho$ is the filling factor. The anisotropy constants $K_1$,$K_2$ chosen are those of pure iron^29^. SMCs come in a wide variety of available particle sizes, they approximately range from $1-150\,\upmu \text {m}$. The following simulations employs particles with $D=1\,\upmu \text {m}$, which places the simulated particles at the lower end of the spectrum. This is necessary in order for the simulation to be numerically feasible since the size of the particle one can simulate is limited by the memory of the GPU at hand. The size of the grains are varied in the study between $10-300\text { nm}$, if not stated explicitly the grain size is $d=140\text { nm}$. Choosing *d* much larger would result in particles consisting of an unrealistically low number of grains, meaning that the seed of directions would influence the result significantly, however choosing the grainsize to small would mean that the random anisotropy model, which will be discussed later, would become relevant. Due to the cubic simulation box, finite difference methods are well suited, which are computationally less expensive compared to finite element methods. Hence, we use our in house developed finite difference code **magnum.np**^13^ for the micromagnetic model. One micromagnetic simulation consists of integrating the Landau-Lifschitz-Gilbert equation for a given geometry and parameters for a duration of three periods $\frac{3}{f}$. Obviously only one period is needed for the evaluation of the powerloss, however since the damping is relatively small, we calculate two periods prior to evaluating so that the system is in a reproducible state. The result is a hysteresis curve $B_x(H_x)$, from which one can evaluate the micromagnetic powerloss $P_m$ and the permeability $\mu _r$. Figure 4 shows a standard hysteresis for $f=1\text { MHz}$ and material parameters as defined in table 1.

**Fig. 5:**
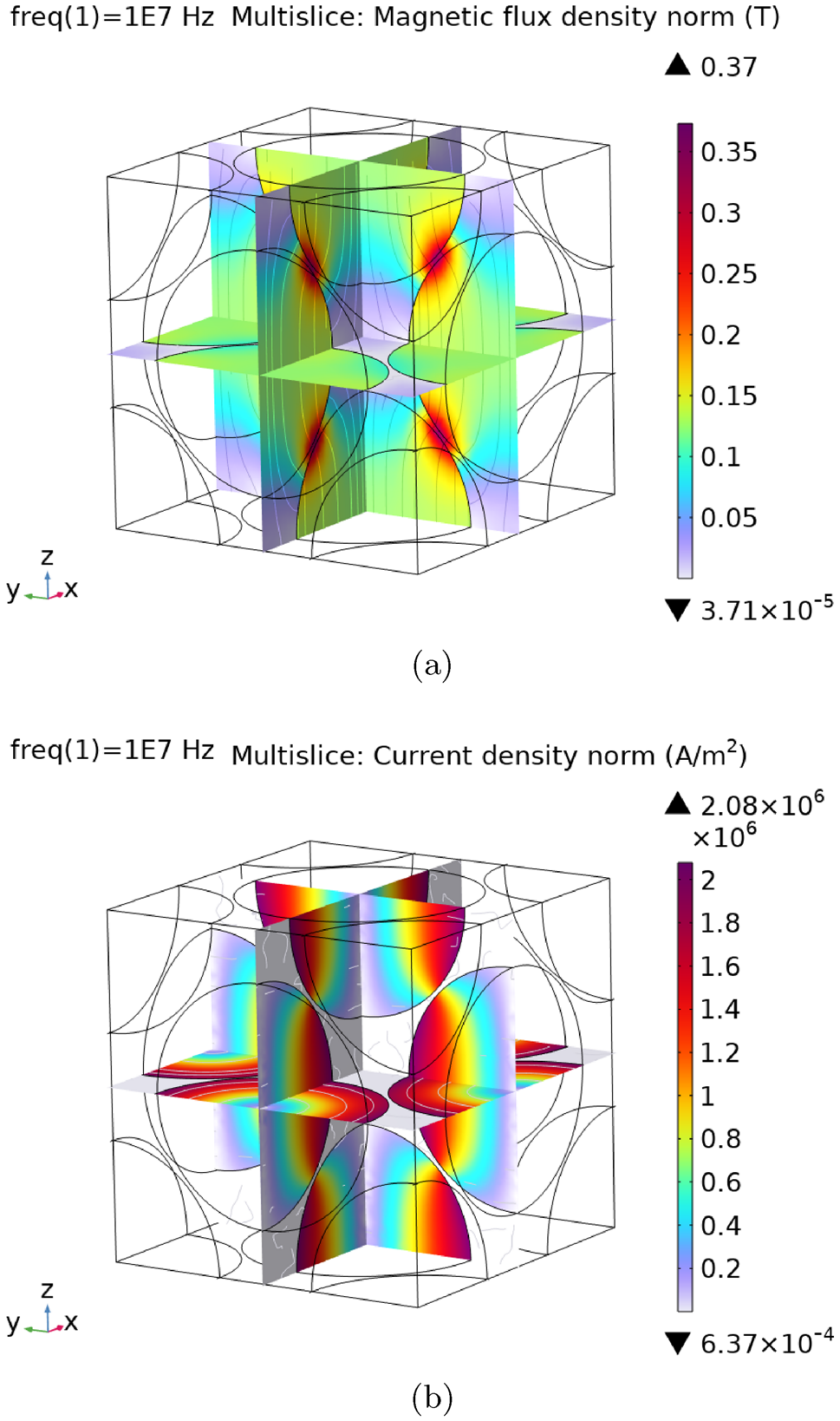
The results of a Maxwell-solver simulation, with only the center fcc unit cell cut out. (**a**) Magnetic flux density distribution $\textbf{B}$ for $f=10\text { MHz}$ & $B=100\text { mT}$. (**b**) Electric current density distribution $\textbf{J}$ for $f=10\text { MHz}$ & $B=100\text { mT}$.

An eddy-current simulation involves solving Maxwell’s equations, as shown in Eq. (8), using the symmetry boundary conditions from Eq. (7) in the frequency domain. We used *Comsol* to carry out these simulations. Figure 5 presents the outcome of a particular simulation with $f=1\text { MHz}$, $B=100\text { mT}$ and the material parameters as shown in Table 2. Specifically, Fig. 5a,b display the norm of the magnetic flux density $|\textbf{B}|$, and the norm of the electric current density $|\textbf{J}|$ as a function of space, respectively. The region of the simulation box shown in the figures represents only the parts which were evaluated to calculate the power loss $P_e$.

**Table 1:** Unless specifically stated otherwise, this table shows the parameters regarding the magnetic and geometric properties of the magnetic particle and the filler material which together forms the entire micromagnetic simulation box.

Constant	Symbol	Value magnetic particle	Value filler	Unit
Saturation magnetization	$M_s$	1.5/$\mu _0$^28^	0	$\text {A/m}$
Cubical anisotropy 1st order	$K_1$	48.0^29^	0	$\text {kJ/m}^{3}$
Cubical anisotropy 2nd order	$K_2$	0.5^29^	0	$\text {kJ/m}^{3}$
Exchange coupling	*A*	10.0	0	$\text {pJ/m}$
Alpha damping	$\alpha$	0.1	0	$\text {a.u.}$
Particle size	*D*	1.0	/	$\upmu \text {m}$
Grain size	*d*	140.0	/	$\text {nm}$
Filling factor	$\rho$	70.0	30.0	$\%$

**Table 2:** Unless specifically stated otherwise, this table shows the parameters regarding the magnetic and geometric properties of the magnetic particles and the filler material which together forms the entire Maxwell-solver simulation box.

Constant	Symbol	Value magnetic particle	Value filler	Unit
Conductivity	$\sigma$	1e6	1	$\text {S/m}$
Permittivity	$\epsilon _r$	$\infty$	1	[a.u.]
Magnetic bulk permeability	$\mu _b$	1e3	1	$\text {[a.u.]}$
Particle size	*D*	1.0	/	$\upmu \text {m}$
Filling factor	$\rho$	70.0	30.0	$\%$

## Results and discussion

### The effect of random grains

The micromagnetic calculations at hand have a random contribution, namely the direction of the anisotropy within the grains, as well as the grain size and shape. Hence, we first show that our statistics is sufficiently good in order to obtain reliable results. This is only necessary for the micromagnetic simulations, since Maxwell equations do not account for anisotropy.

**Fig. 6:**
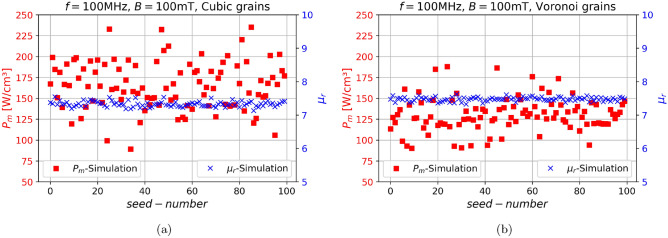
The magnetic powerloss $P_m$, and the permeability $\mu _r$ of the micromagnetic SMC-simulation models utilizing an fcc-particle-structure with differing (**a**) directional seeds in cubic grains. (**b**) Voronoi structures in Voronoi grains.

For this matter, we perform 100 simulations with different directional seed for each calculation, while keeping the total number of cubic grains constant. The frequency was chosen to be $f=100\text { MHz}$, since we care only about the statistics, we increased the frequency, which results in significantly reduced simulation time. Figure 6a along with Fig. 6b shows the results of the aforementioned study. The permeability $\mu _r$ seems to be completely independent with $\mu _r=7.39 \pm 0.06$, however the powerloss $P_m$ does have a dependence on the directional seed with $P_m=(166.8 \pm 30.0)\text { kW/cm}^{3}$ . Since a standard deviation of 18% is accurate enough for a first study, the following simulations are performed only with a single seed. However, if very small deviations are investigated the statistic has to be improved for more accurate results. A similar test was carried out with the Voronoi grains where we again performed 100 simulations where only the grain structure, was changed while the seed direction remained the same with each simulation, each time with a different seed of new centers which always gives a different Voronoi-structure, as is demonstrated in Fig. 6b. Again we observe that the permeability is $\mu _r=7.47 \pm 0.06$, meaning that the permeability is rather independent on the Voronoi seed, and also that it does not matter for the permeability wether regular or Voronoi grains are used. Examining the powerloss with Voronoi grains shows $P_m=(130.1 \pm 19.9)\text { kW/cm}^{3}$, this means that different structures result in a smaller deviation than different directional seeds. However since both models show qualitatively similar results, in the following study the regular grain model is used. This has the additional advantage that they are easier to scale in terms of grainsize *d*.

**Fig. 7:**
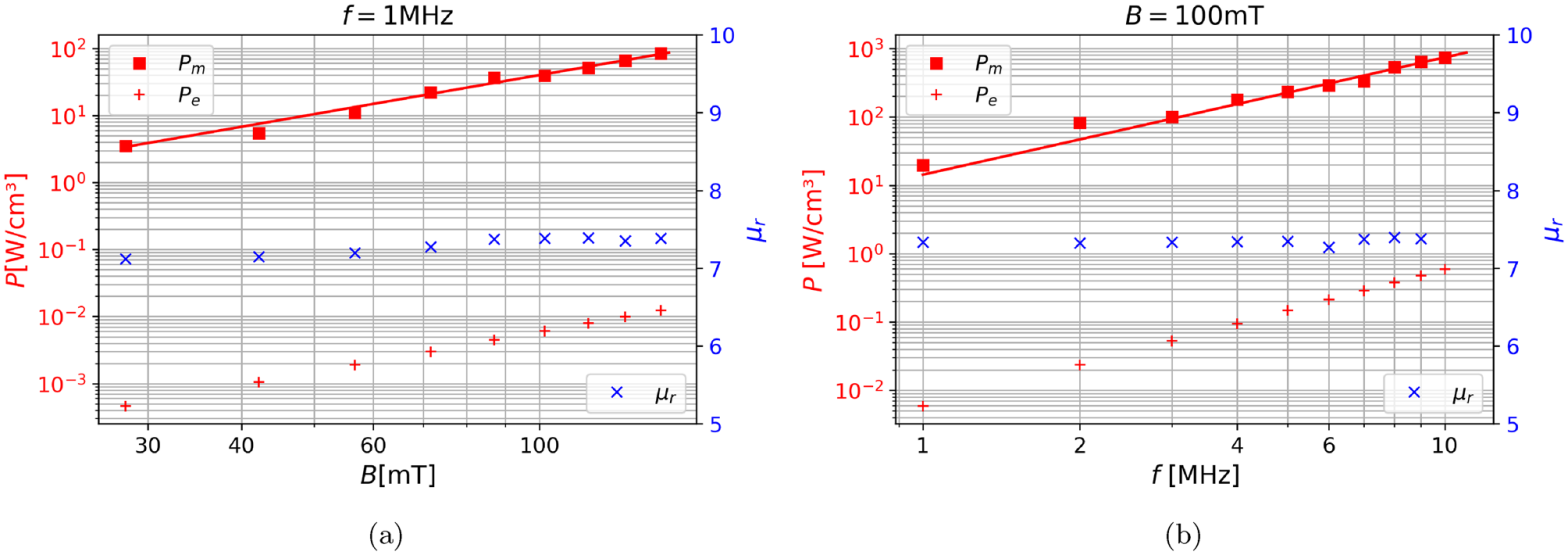
The magnetic powerloss $P_m$, the eddy-current powerloss $P_e$ and the permeability $\mu _r$ of the two decoupled SMC-simulation models utilizing an fcc-particle-structure and cubic grains for a sweep over (**a**) the magnetic flux density *B* at $f=1\text { MHz}$. (**b**) the frequency *f* at $B=100\text { mT}$.

### Sweep over magnetic flux density and frequency

The classic sweeps by which the magnetic losses of SMCs are characterized are the dependencies on the frequency *f* and the magnetic flux density *B*. This is represented by a phenomenological equation which is known as the Steinmetz equation

10 $$\begin{aligned} P_m=kB^{\alpha }f^{\beta }, \end{aligned}$$

where *k*, $\alpha$ and $\beta$ are the Steinmetz coefficients, which are material parameters, generally calculated from the hysteresis by fitting. It is well known that the eddy-current losses scale with $B^2$ and $f^2$. Figure 7 shows the sweeps over magnetic flux density *B* and frequency *f* for the magnetic powerloss $P_m$, the eddy current powerloss $P_e$ and the permeability $\mu _r$. $\mu _r$ remains almost completely unchanged with increasing *B* or *f*, which is also what is to be expected according to literature^9,30^. The trends for $P_m$ show a linear increase in the log-log plot which means that they increase exponentially, through fitting we determined that the Steinmetz coefficient are $\alpha =1.93$, $\beta =1.71$, as is expected from the literature^31^. This gives validity to our model, since the simulations obeys Steinmetz equation, along with the Steinmetz coefficients being in the correct range.

From the log-log plot we can easily determine that the eddy-current losses increase with $f^2$ and $B^2$, moreover we can see $P_e$ is multiple magnitudes smaller than $P_m$. Therefore according to our model the magnetic losses, meaning the static hysteresis losses as well as the dynamic losses, which add on to the former, are significantly larger than $P_e$. This is not entirely surprising since SMCs are designed toward having very low $P_e$. Therefore there is no reason to further optimize these materials to lower $P_e$, but only to optimize towards decreasing $P_m$.

### Sweep over anisotropy constant

**Fig. 8:**
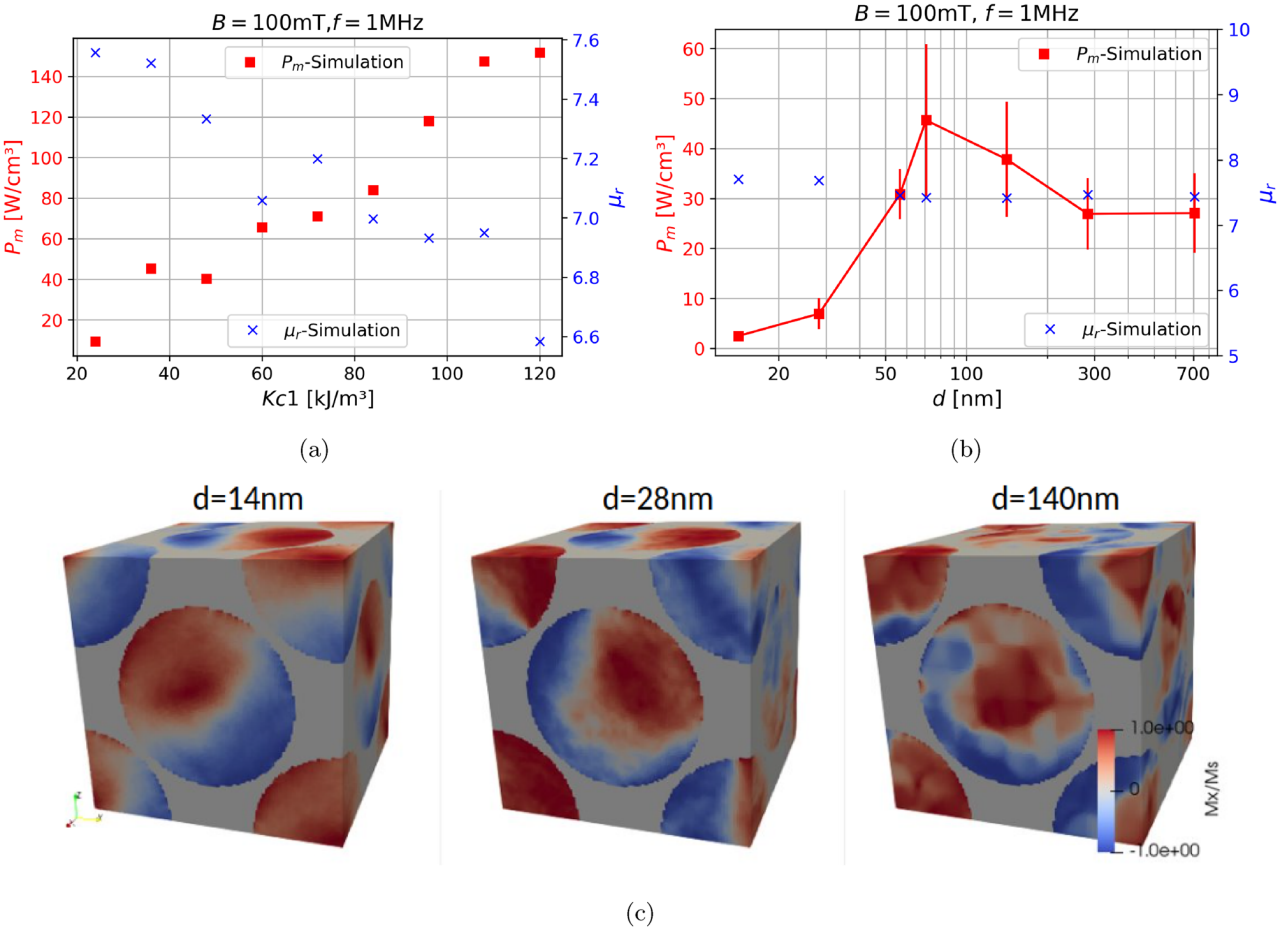
(**a**) Sweep over the anisotropy constant *Kc*1 is shown, for the micromagnetic powerloss $P_m$ and the permeability $\mu _r$. (**b**)Simulating the random anisotropy model, by changing the grain-size *d* for the micromagnetic powerloss $P_m$ and the relative permeability $\mu _r$, along with (**c**) the magnetization images at $B=100\text {mT}$.

A parameter which is crucial to the performance of SMCs is the crystal anisotropy constant $K_1$. However the influence of $K_1$ on the power-losses and the permeability has up to now hardly been investigated at all. This is is in part due to the fact that the measurement of $K_1$ is rather difficult for polycrystalline samples. This is where simulations as a tool can become helpful, since the anisotropy constant is one of the input parameters of the micromagnetic simulations. Figure 8a shows the losses $P_m$ and the permeability $\mu _r$ as function of the anisotropy constant $K_1$, for $B=100\text {mT}$ and $f=1\text { MHz}$. It is evident that an increase in $K_1$ causes an increase in $P_m$ while it slightly decreases the permeability $\mu _r$. The reason $K_1$ only slightly changes the permeability is, that the air gaps between the magnetic sphere dominate the effective permeability of the entire structure. The increased power-loss is intuitively clear since higher anisotropies result in more irreversible switching processes, which leads to increased hysteresis and therefore higher power-loss.

### Design concepts aimed toward improving magnetic performance

#### The random anisotropy model

Based on the results of the previous section one might conclude that to produce a better softmagnet one simply has to use a material with a small or vanishing crystal anisotropy $K_1$. While this would certainly be a solution, an easier one might be to decrease the grainsize *d*, therefore utilizing the random anisotropy model. The random-anisotropy model was first proposed by Herzer^32^. To understand it one needs to understand that the critical scale at which the exchange energy balances the anisotropy energy is called the ferromagnetic exchange length $L_0$, which, in the case of uniaxial anisotropy, can be written as

11 $$\begin{aligned} L_0=\sqrt{A/K_{u1}}, \end{aligned}$$

$L_0$ is the minimum length at which the magnetization can change in direction and therefore also determines the domainwall-width, *A* is the exchange constant and $K_{u1}$ is the uniaxial anisotropy constant. If one were to reduce the grainsize *d* below the exchange length $L_0$, the magnetization will not follow the direction of the individual grains, but instead is forced to align parallel by the exchange interaction. Therefore the effective anisotropy is averaged over several grains and thus considerably reduced in magnitude. The difference this makes for the coercive field $H_c$ and therefore also for the powerloss in the experiment can be observed in Ref.^32^. We have performed the same parameter-study with our simulations by reducing the grainsize in our model, as shown in Fig. 3A. The grains in our model are all equally sized cubes, meaning that decreasing *d* decreases the cubes until they get to the extreme where every single simulation cell is a different grain. The calculated results are illustrated in Fig. 8b, along with the magnetization configuration at $B=100\,mT$ for different grainsizes in Fig. 8c. $P_m$ increases with increasing *d*, due to the fact that the effective anisotropy is decreased, until it plateaus at $d\approx 70\text { nm}$, and then decreases again, reflecting the more conventional rule that good soft magnetic properties require large grains^33^. This behavior was already observed experimentally^32,34^. The permeability remains completely unaffected by the change in grainsize, which is not what one would expect from Ref.^32^. This discrepancy stems from the difference in hysteresis shapes between^32^ and our calculations. The SMCs we simulate can be characterized as airgap-dominated magnetic composites, since as already shown in Ref.^8^ the hysteresis depends heavily on the airgap, since it gets more and more sheared the bigger the airgap is or in other words, the smaller the fill-factor becomes. For small airgaps the hysteresis looks rectangular and the coercive field $H_c$ is directly related to the permeability $\mu _r \propto H_c^{-1}$. This does not hold for hysteresis which are more sheared, as they are in our calculations due to the smaller fill-factor.

#### Reducing hysteresis loss under transverse magnetic field

Feng et al.^30^ have proposed a method with which the power loss of SMCs decreases at the cost of reducing the permeability to a lesser extent. It was found that if a transverse magnetic field is applied to the SMC core the power loss reduced by about 1/3 at the expense of less than 10% decrease in permeability. The effect is explained by a reduction of the hysteresis loss which is attributed to spin rotations and domain wall displacements which is completely locked in the direction of the applied field. Since this method effects the hysteresis loss, the proposed micromagnetic model should be able to replicate this effect.

**Fig. 9:**
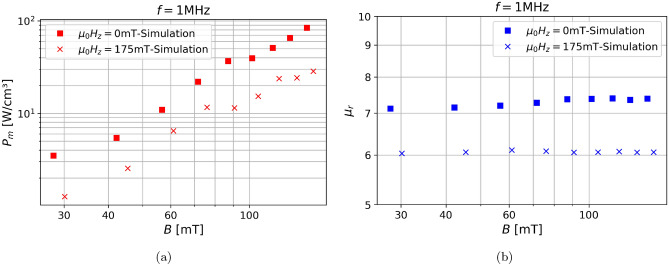
(**a**)The powerloss $P_m$ and (**b**) the permeability $\mu _r$ are shown for different magnetic flux densities *B* with and without applying a transversal field $\mu _0 H_{trans}=175\text { mT}$.

To replicate the condition of the experiment we have repeated the simulations from Fig. 7a but this time we have applied a constant magnetic field in the z-direction with $|\mu _0 \textbf{H}_{trans}|=175\text { mT}$ while the AC-field is applied in the x-direction. Figure 9 shows the comparison between the losses of the performance of the model with and without a transversal field. The results show that the overall trend is the same in the simulation and in Ref.^30^. However in detail the trends differ, the simulations show a decrease in the powerloss by factor of roughly 4 while the permeability decreases by 20%. This discrepancy can be explained by the fact that in contrast to our simulations the experiments in Ref.^30^ were conducted in the kHz regime, therefore one could call them quasistatic and the losses given are simply the hysteresis losses, in contrast, we simulate the dynamic loss.

#### Comparison with experiments

**Fig. 10:**
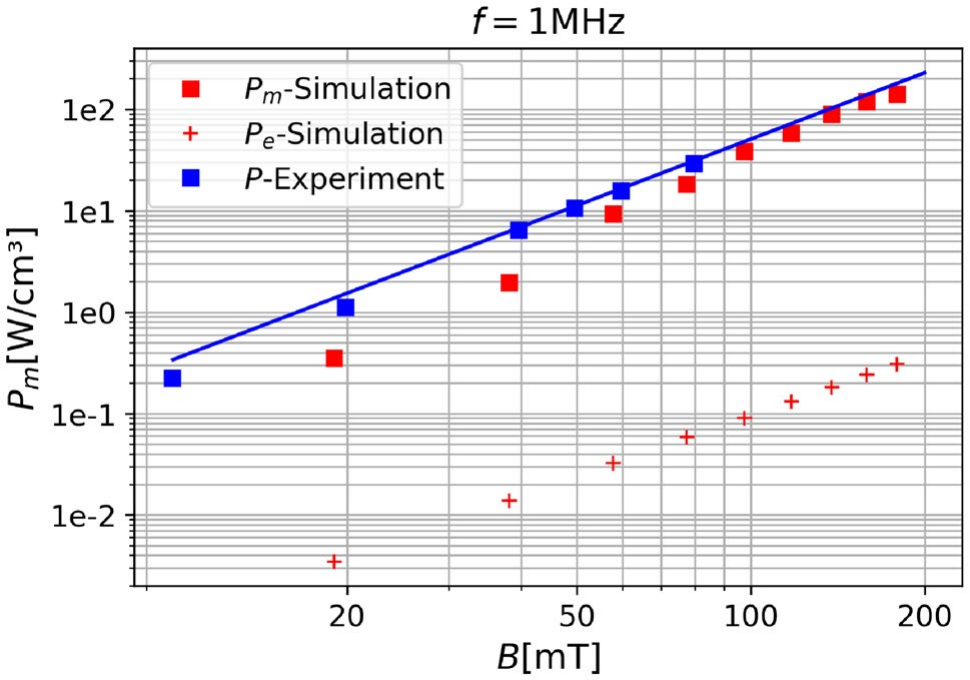
The comparison between the measured and the simulated powerloss for a Carbonyl iron powder^35^ is shown as a function of the magnetic flux density *B*.

In order to compare our findings to experimental results, we have performed measurements of Carbonyl iron powder^35^. SMC samples were prepared by mixing insulated soft magnetic carbonyl iron powder of grade SWS-S from BASF (median particle diameter $D_{50} = 3.8\, {\upmu }\text {m}$) with an epoxy resin. The mixture was then molded into toroidal geometries under uniaxial compaction at a pressure below $30 \text { MPa}$. This low-pressure approach was chosen to avoid mechanical deformation of the iron particles and to avoid the introduction of internal stresses that could influence the magnetic performance. After molding, the samples were thermally cured and demolded without further post-processing. The magnetic properties of the toroidal samples were analyzed using an IWATSU SY-8218 B-H analyzer. All measurements were carried out under purely alternating current excitation at a fixed frequency of 1 MHz. To evaluate the field-dependent magnetic behavior and associated losses, magnetic flux density sweeps were performed. The use of toroidal ring cores ensured a closed magnetic path, minimizing demagnetizing effects and stray fields during measurement. For the purpose of comparison, this means that certain simulation parameters need to be altered from Tables 1 and 2 to that of pure iron, as can be seen in Table 3, all parameters not explicitly mentioned therein were taken from Tables 1 and 2. Furthermore we have seen that the directional seed influences the outcome of the micromagnetic simulation. Therefore the experimental results will be compared to micromagnetic simulations which have been carried out for ten different directional seeds and averaged afterwards, this is not necessary for the eddy-current simulation, since it doesn’t have a random contribution. Figure 10 shows the results of this comparison. All results have been averaged over the multiple measurements or simulations which were carried out. Since there are only few measuring points available, a fit was drawn through the data so that it is more easily comparable. We see that the simulated data is below the experimental data. This discrepancy decreases at higher field and is at its highest at lower fields. Adding the two simulated energy contribution would hardly change the result since the eddy-current losses are orders of magnitude below the micromagnetic losses, which is to be expected. One reason for this discrepancy could be an additional loss-source, which is not in the scope of this work. Anomalous eddy current losses are distinct from classical eddy current losses and are caused by the movement of magnetic domain walls within the material^36^. The losses would add onto our calculated loss and might explain the gap between the simulated and the experimental results. Calculating these losses would require to couple the micromagnetic model with the Maxwell model, which is outside the scope of this work. However this is not necessarily an issue. The simulations show good enough agreement and correctly predict the slope as a function of the magnetic flux density *B*.

**Table 3:** This table shows the parameters used for the simulation of iron-powder, which were performed with the aim to compare the results to the measurements.

Symbol	Value magnetic particle	Unit
$M_s$	2.2/$\mu _0$^28^	A/m
*A*	22.0	pJ/m
$\alpha$	0.01	a.u.
*D*	4.0	$\upmu$m
*d*	140.0	nm

## Conclusion

We have presented a numerical model, which allows to simulate soft magnetic materials without finite size effects. This was accomplished through the implementation of periodic boundary conditions within the strayfield computation. All relevant energy contributions were demonstrated to be calculable using two distinct simulation models: the first based on micromagnetism and the second solving Maxwell’s equations. We have chosen to verify our results by reproducing experiments which aim to alter the powerloss or the permeability. The simulations obey Steinmetz-law when it comes to the *B* and *f* dependent powerloss $P_m$, with the Steinmetz-coefficients being $\alpha =1.93$ and $\beta =1.71$. It was shown that the powerloss can be reduced by utilizing the random anisotropy model through decreasing the grainsize *d*. Alternatively the powerloss can be decreased by applying a transversal field $H_{trans}$, while at the same time decreasing the permeability by a lesser degree. Additionally, we compared our simulations to experimental findings. While the results did not match perfectly, the fact that they are within the same order of magnitude is highly encouraging. Furthermore, we have demonstrated how power loss is distributed among various loss contributions-an analysis rarely conducted, particularly for frequencies in the MHz range. Notably, our findings reveal that micromagnetic losses are the dominant factor, highlighting the importance of focusing future development efforts on minimizing these losses. The financial support of Huawei is acknowledged.

## Data Availability

The datasets used and/or analyzed during the current study is available from the corresponding author on reasonable request.
